# Dr. Ephraim Winnie

**Published:** 1913-04

**Authors:** 


Dr. Ephraim Winnie of Hornell, University of Michigan 1857, died at Hornell, Feb. 19.

